# Optic disc parameters and choroidal vascular index as potential risk indicators in non-arteritic anterior ischaemic optic neuropathy: a retrospective study

**DOI:** 10.7717/peerj.20695

**Published:** 2026-01-28

**Authors:** Emine Atalay, Abdullah Beyoğlu

**Affiliations:** 1 Ophtalmology, Gaziantep City Hospital, Gaziantep, Turkey; 2 Ophtalmology, Selcuk University, Konya, Turkey

**Keywords:** Optic disc, NAION, OCTA, Choroidal vascular ındex, RNFL

## Abstract

**Background:**

Although previous research has explored the involvement of the choroid in the pathogenesis of non- arteritic anterior ischaemic optic neuropathy (NAION), the relationship between optical coherence tomography angiography (OCTA) findings and choroidal features remains unclear. An understanding of this relationship may help clarify the vascular mechanisms underlying this disease. The aim of this study was to investigate the relationships between OCTA and choroidal parameters in patients with NAION during the post-acute phase, after the resolution of optic disc oedema.

**Methods:**

This retrospective analysis included the affected eyes of patients with unilateral NAION, their unaffected fellow eyes, and the eyes of age- and sex-matched healthy controls. The three groups were compared with regard to OCTA and choroidal parameters. Retinal imaging was conducted approximately 2 months after NAION occurrence to allow for the spontaneous resolution of characteristic optic disc oedema.

**Results:**

A total of 75 eyes were included in the final analysis: 25 NAION-affected eyes, 25 fellow eyes, and 25 control eyes (13 women and 12 men). Age and sex distributions were similar across groups. The peripapillary vessel density (pVD), flow area (FA), retinal nerve fibre layer (RNFL) thickness, and choroidal vascularity index (CVI) in all quadrants were significantly lower in NAION eyes than in unaffected and control eyes. Unaffected eyes also demonstrated significantly lower radial peripapillary capillary (RPC) mean, RPC temporal, and RPC FA values than did the healthy controls. A moderate correlation was observed between RPC pVD and the mean RNFL thickness in NAION eyes and between RPC FA and the mean RNFL thickness in both NAION and unaffected eyes. The strong relationship between RPC perfusion and RNFL thinning could not be statistically confirmed after false discovery rate correction; thus, a direct cause-and-effect relationship could not be validated.

**Conclusions:**

There were no significant correlations between OCTA and choroidal parameters across all groups. These findings suggest that the retina and choroid are affected through distinct mechanisms in NAION. However, reductions in OCTA parameters, including CVI, were evident in NAION eyes. Overall, the study findings underscore the potential of OCTA as a non-invasive tool for identifying risk factors and monitoring disease progression in NAION.

## Introduction

Non-arteritic ischaemic optic neuropathy (NAION) is the most common cause of non-glaucomatous optic neuropathy in middle-aged and older adults. It typically presents with painless vision loss, optic disc oedema, and visual defects. Ischaemia plays a central role, although the exact aetiology remains unclear. Previous studies have described the vascular dysregulation and optic nerve head (ONH) perfusion deficits underlying the pathophysiology of NAION (Hayreh, 2011; Patil, Biousse & Newman, 2022). In this study, we evaluated the post-acute clinical phases (after resolution of optic disc oedema) with emphasis on anatomical and vascular degradation following acute ischaemic insult.

Optical coherence tomography (OCT) angiography (OCTA) is increasingly used to examine retinal and choroidal changes in conditions such as NAION, where vascular pathology plays a key role (Spaide et al., 2018).

NAION has been associated with occlusion of the short posterior ciliary arteries, which supply blood to the peripapillary choroid and contribute to the blood supply to ONH. Consequently, impaired perfusion of the peripapillary choroid may contribute to NAION development. Recent studies have suggested that the choroid may be involved in various optic nerve disc pathologies. The choroidal vascularity index (CVI) has been proposed as a reliable parameter of choroidal vascularity (Agrawal et al., 2020; Iovino et al., 2020). It represents the luminal vascular ratio of the choroid on binary OCT images; variations in image thresholds might alter measurement outcomes; thus, intra-study comparators remain highly significant (Sonoda et al., 2014).

Assessment of the correlation between the retina and choroid may be important to assess disease progression and identify potential risk factors. The aim of this study was to investigate the mechanisms underlying vascular changes in diseases such as NAION by examining the relationship between retinal and choroidal parameters and improving disease monitoring. We also explored whether optic disc and choroidal parameters serve as potential risk indicators in NAION. There is a lack of comprehensive data regarding the relationship between optic disc parameters and choroidal measurements in previous studies using OCTA; the present study aimed to address this gap in the literature.

## Materials & Methods

This retrospective, comparative, observational study included patients who presented to the Department of Ophthalmology at Kahramanmaras Sutcu Imam University Hospital between September 2019 and September 2022. The hospital, a tertiary healthcare centre with advanced diagnostic and examination facilities, was selected because of the feasibility of diagnosing NAION and conducting regular patient follow-ups. Patients with sudden, painless, unilateral vision loss; relative afferent pupillary defects; optic disc oedema; altitudinal visual field defects; normal erythrocyte sedimentation rate/C-reactive protein levels; no signs of giant cell arteritis; and normal neurological findings on imaging were diagnosed with NAION. All patients diagnosed with NAION between September 2019 and September 2022 were retrospectively identified from hospital records, and those who met the inclusion and exclusion criteria were consecutively enrolled in the study. No randomization was performed; all eligible participants were included without selection bias. The data used for analysis were obtained after disc oedema had resolved (average 2 months) in NAION eyes.

This study was approved by the Institutional Review Board (IRB) of Kahramanmaras Sütçü Imam University, Faculty of Medicine (Decision No: 01, Date: 09/11/2022) and conducted in accordance with the Declaration of Helsinki. Because of the retrospective, non-interventional nature of the study and the use of anonymized data, the requirement for informed consent was formally waived by the IRB.

The sample size was calculated using G*Power software on the basis of data from a comparable study in the literature. Power analysis indicated that with a significance level of *α* = 0.05 and statistical power of 80% (*β* = 0.20), an effect size of 0.79 would require a minimum of 25 eyes per group. Accordingly, 25 patients with complete data, confirmed unilateral involvement, and no exclusion criteria were enrolled. Both the affected and unaffected eyes of these patients were included in the unilateral NAION group. We excluded individuals with bilateral optic disc oedema, retinopathy, glaucoma, other optic disc pathologies, a history of ocular surgery, or refractive error outside the ±3D range. The control group comprised 25 age- and sex-matched healthy individuals who had undergone OCTA as part of a routine examination and did not have any ocular pathologies. These participants were matched to patients with NAION within a ±2 year age range and by sex to minimise potential confounding. Data for the NAION eyes and the unaffected eyes of the 25 patients were compared with the data for the right eyes of the 25 healthy individuals (right eyes).

For the NAION, unaffected, and control eyes, OCTA images were subjected to rigorous quality control. Images with a signal strength index below 50, abnormal or unreliable segmentation, motion artefacts, or poor centration were excluded. Furthermore, rigorous attention was paid to manual review and correction of segmentation artifacts in all remaining images, a critical step to ensure that peripapillary measures, particularly for eyes with less common anatomical structures, were not disproportionately altered (Campbell et al., 2017).

**Figure 1:**
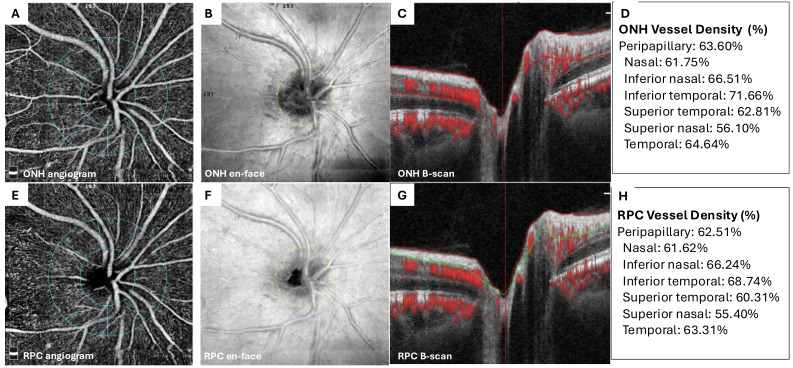
Representative multimodal optical coherence tomography angiography (OCTA) images of a healthy control eye. (A and E) demonstrate the automated segmentation of the 4.50-mm peripapillary region for the optic nerve head (ONH) and radial peripapillary capillary (RPC) layers. Corresponding multimodal OCTA outputs are shown for each layer, with en face projections in (B and F) and structural B-scan images in (C and G). Vessel density values displayed in (D and H) represent the automated measurements generated by the OCTA device and include both global and sector-specific parameters. (A–D) (ONH); (E–H) (RPC).

Follow-up examination data and OCTA parameters were analysed. All OCTA measurements were performed using the same device and standardised protocols to minimise measurement bias. OCTA images were acquired with the AngioVue software on the RTVue-XR device (Optovue Inc., Fremont, CA, USA). Quantitative analyses included measurement of the peripapillary vessel density (pVD), flow area (FA), and retinal nerve fibre layer (RNFL) thickness. Representative images from a healthy control eye are shown in Fig. 1, which illustrates the ONH and radial peripapillary capillary (RPC) layers with automatic segmentation, en face angiograms, and quadrant-specific vessel density values used in the study (Fig. 1).

**Figure 2:**
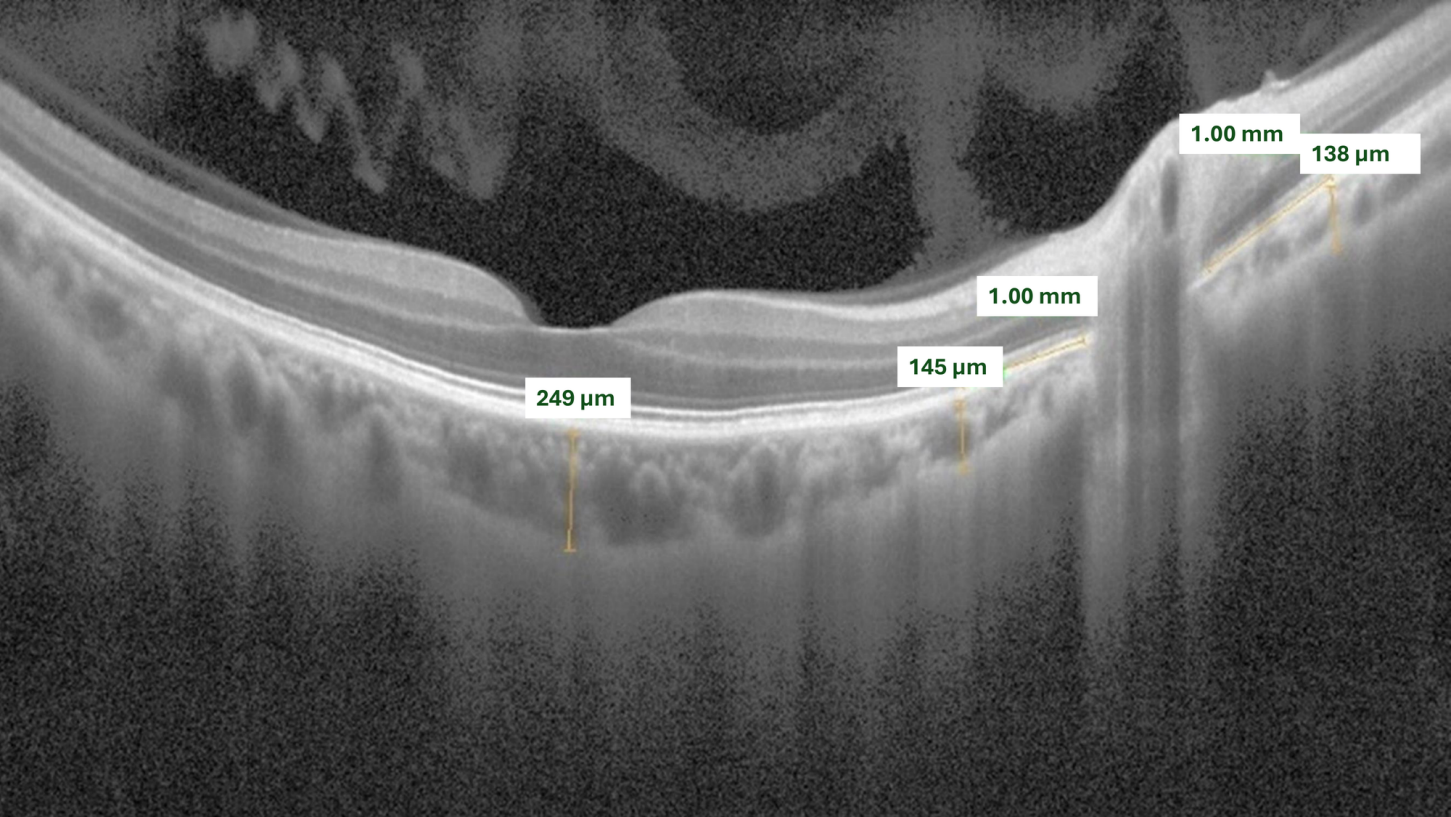
Measuring choroidal thickness. Enhanced depth imaging (EDI) was used to obtain horizontal B-scans passing through the fovea and the optic disc. Choroidal thickness was measured manually as the perpendicular distance between the outer border of the retinal pigment epithelium (RPE) and the inner border of the sclera. Measurements were taken at three predefined locations: Subfoveal, 1,000 µm temporal to the optic disc, and 1,000 µm nasal to the optic disc. The average of these values was used for analysis.

Using enhanced HD line OCTA images, the choroidal thickness (ChT) was calculated by drawing lines at three locations—temporal, nasal, and subfoveal—from the optic disc to the choroid-scleral junction at a distance of 1,000 µm. The same reference points between the retinal pigment epithelium and the choroid–sclera junction using the device’s built-in software (Fig. 2).

**Figure 3:**
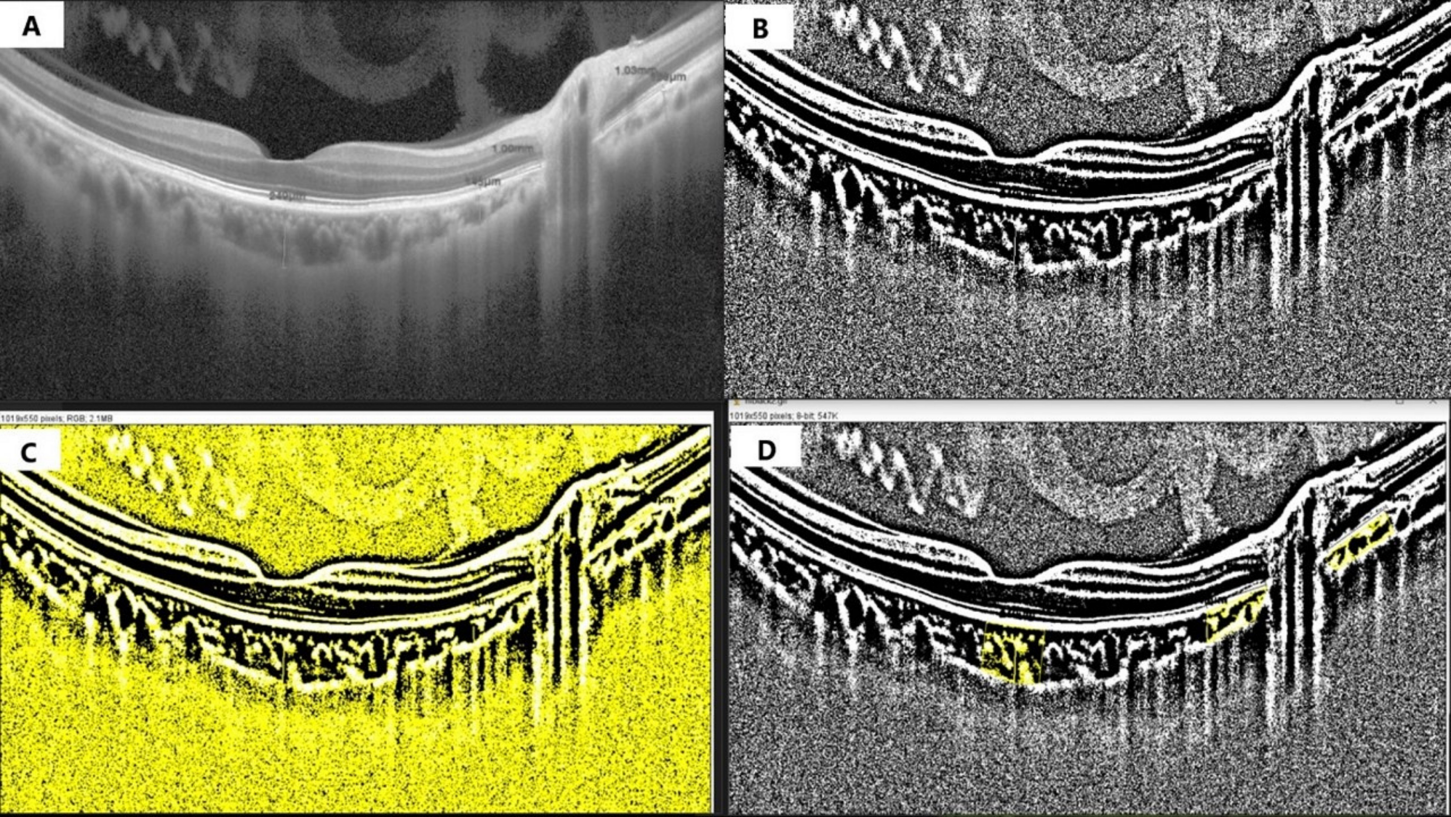
Calculation of choroidal vascularity index. The original EDI-OCT image was converted to 8-bit format for standardization. (B) The image was binarized using Niblack’s auto local threshold, separating the dark (vascular/luminal) and light (stromal) areas. (C) The binarized image was converted to RGB color mode, and the dark pixels representing the luminal (vascular) area were selected. (D) Using the region of interest (ROI) manager tool, both the luminal area (LA) and total choroidal area (TCA) were quantified, and CVI was calculated as LA/TCA ×100.

CVI was measured using the enhanced HD line scans using ImageJ software (version 1.53s), based on a previously described semi-automated protocol. The total choroidal area (TCA) was calculated by measuring the area of all regions drawn. The lumen area (LA) was determined using software based on the dark pixels in the segmented regions. The stromal area (SA) was calculated by subtracting LA from TCA. CVI was then calculated by dividing the LA by the TCA (Fig. 3).

All OCTA images were obtained with high signal strength, with no motion or segmentation artifacts. Because the data were semi-automatically acquired, a minimal degree of measurement error was considered inevitable. To minimize this potential variability, only images of the best quality were included, and all analyses were performed by a single experienced examiner under identical conditions. Two separate scans were processed for each eye, and the mean value of the two measurements was recorded as the final data point. Measurements were performed on the basis of predefined anatomical reference points to ensure internal consistency across repeated evaluations. Previous studies using similar OCTA-based CVI and ChT measurement protocols have demonstrated high reproducibility and supported the reliability of this technique (Agrawal et al., 2017; Malamas et al., 2019; Pellegrini et al., 2019).

In this study, quantitative variables were analysed as continuous data, and descriptive statistics included the mean, standard deviation, median, minimum, maximum, frequency, and ratio. The Kolmogorov–Smirnov test was used to assess the distribution of variables. For independent quantitative variables, the independent samples *t*-test and Mann–Whitney U test were applied, depending on data normality. Paired sample t-tests and Wilcoxon tests were used for dependent quantitative variables. The chi-square test was used to analyse independent qualitative variables. Spearman’s rank correlation was used to evaluate the relationships between parameters within each group. Effect sizes for group comparisons were calculated as Cohen’s d with 95% confidence intervals (CIs). Effect sizes of 0.2, 0.5, and 0.8 were interpreted as small, medium, and large, respectively. All analyses were conducted using SPSS version 28.0, with statistical significance set at *P* < 0.05. The Bonferroni correction was applied to control the Type I error rate from multiple comparisons. After correction, results with *P*-values < 0.0167 were considered statistically significant.

## Results

A total of 75 eyes were included in the final analysis: 25 NAION-affected eyes, 25 fellow eyes, and 25 control eyes (13 women and 12 men). All participants had complete and high-quality OCT and OCTA scans suitable for analysis. No participants were excluded because of poor image quality or missing data. The mean age was 59.1 ± 10.3 years in the NAION group and 58.6 ± 9.7 years in the control group. There were no statistically significant differences in age and sex distribution between the NAION (affected and unaffected) and control groups (Table 1).

**Table 1:** Peripapillary vessel density, flow area and retinal nerve fibre layer thickness.

	**NAION eyes (*n* = 25)**	**Unaffected eyes (*n* = 25)**	**Control eyes (*n* = 25)**	***P* value** **NAION *vs* Control**	***P* value** **NAION *vs* Unaffected**	***P*value** **Unaffected *vs* Control**
Mean age	59.1 ± 10.3		58.6 ± 9.7	0.866^*^		
Male sex N(%)	12 (%48)		12 (%48)	1		
Mean ONH pVD (%)	46.0 ± 7.9	60.3 ± 3.7	62.0 ± 2.7	<0.001^*^	**<0.001^a^**	0.055^*^
Nasal ONH pVD (%)	45.7 ± 8.3	59.6 ± 3.2	60.6 ± 3.7	<0.001^*^	**<0.001^a^**	0.362^*^
İnferionasal ONH pVD (%)	50.2 ± 12.1	61.9 ± 5.7	64.2 ± 3.0	<0.001^*^	**<0.001^a^**	0.265^*^
İnferiotemporal ONH pVD (%)	44.2 ± 10.2	62.4 ± 5.2	64.3 ± 3.7	<0.001^*^	**<0.001^a^**	0.160^*^
Süperionasal ONH pVD (%)	44.1 ± 10.9	59.4 ± 6.2	62.3 ± 4.4	<0.001^*^	**<0.001^a^**	0.133^*^
Süperiotemporal ONH pVD %	48.5 ± 10.9	63.4 ± 4.7	65.9 ± 3.2	<0.001^*^	**<0.001^a^**	0.045 ^*^ns
Temporal ONH pVD (%)	45.0 ± 10.1	58.3 ± 6.8	61.2 ± 5.6	<0.001^*^	**<0.001^a^**	0.081^*^
Mean RPC pVD (%)	43.2 ± 8.3	61.3 ± 3.6	64.0 ± 3.7	<0.001^*^	**<0.001^a^**	0.011^*^
Nasal RPC pVD (%)	43.3 ± 9.4	60.0 ± 3.2	60.6 ± 4.7	<0.001^*^	**<0.001^a^**	0.372^*^
İnferionasal RPC pVD (%)	47.7 ± 13.0	63.5 ± 6.0	65.7 ± 3.2	<0.001^*^	**<0.001^a^**	0.455^*^
İnferiotemporal RPC pVD (%)	45.8 ± 13.0	66.1 ± 4.6	68.8 ± 3.0	<0.001^*^	**<0.001^a^**	0.037 ^*^ns
Süperionasal RPC pVD (%)	41.4 ± 10.0	64.5 ± 5.6	67.2 ± 4.3	<0.001^*^	**<0.001^a^**	0.054^*^
Süperiotemporal RPC pVD (%)	41.8 ± 11.3	59.5 ± 6.2	63.4 ± 5.1	<0.001^*^	**<0.001^a^**	0.025 ^*^ ns
Temporal RPC pVD (%)	41.3 ± 9.6	58.9 ± 6.0	64.7 ± 5.2	<0.001^*^	**<0.001^a^**	<0.001^*^
ONH FA (μm^2^)	1,386 ± 179	1,635 ± 90	1,651 ± 171	<0.001^*^	**<0.001^a^**	0.076^*^
RPC FA (μm^2^)	1,210 ± 252	1,420 ± 210	1,608 ± 197	**<0.002^c^**	**<0.001^b^**	**0.002^c^**
Mean RNFL (μm)	72.4 ± 20.4	103.0 ± 7.3	106.1 ± 14.4	<0.001^*^	**<0.001^a^**	0.142^*^
Süperior RNFL (μm)	84.5 ± 29.5	125.7 ± 11.2	131.1 ± 20.7	<0.001^*^	**<0.001^a^**	0.153^*^
Nasal RNFL(μm)	62.8 ± 17.2	82.5 ± 7.2	85.5 ± 19.0	<0.001^*^	**<0.001^a^**	0.992^*^
İnferior RNFL (μm)	90.6 ± 29.5	127.2 ± 11.3	128.2 ± 21.3	<0.001^*^	**<0.001^a^**	0.614^*^
Temporal RNFL (μm)	51.7 ± 17.6	77.0 ± 8.2	79.6 ± 13.3	<0.001^*^	**<0.001^a^**	0.393^*^

**Notes.**

* Mann–Whitney *U* test.

a Wilcoxon test.

c Independent sample *t*-test.

b Paired sample *t*-test.

Statistical significance was set at Bonferroni-adjusted *α* = 0.0167. *P* values < 0.0167 were considered significant.

ns not significant after correction NAION non-arteritic ischemic optic neuropathy FA flow area pVD peripapillary vessel density ONH optic nerve head RPC radial peripapillary capillary RNFL retinal nerve fibre layer thickness

Bold values indicate statistically significant differences (*p* < 0.05).

pVD, FA, and RNFL thickness values in all quadrants were significantly lower for NAION eyes than for unaffected and control eyes. Unaffected eyes exhibited significantly lower RPC mean, RPC temporal, and RPC FA values than did healthy control eyes (*P* = 0.011, *P* < 0.001, and *P* = 0.002, respectively). There were no significant differences in ONH pVD, ONH FA, and RNFL thickness between unaffected and control eyes (Table 1).

NAION eyes showed significantly lower CVI in the temporal, nasal, and subfoveal regions than did the unaffected and control eyes. However, there were no significant differences in temporal, nasal, and subfoveal ChT between NAION, unaffected, and control eyes. In addition, there were no significant differences in CVI and ChT between unaffected and control eyes (Table 2).

**Table 2:** Choroidal thickness and choroidal vascularity index.

	**NAION eyes (*n* = 25)**	**Unaffected eyes (*n* = 25)**	**Control eyes (*n* = 25)**	***P* value** **NAION *vs* Control**	***P* value** **NAION *vs* Unaffected**	***P* value** **Unaffected *vs* Control**
Temporal ChT (μm)	192.4 ± 96.3	196.3 ± 85.4	178.9 ± 51.5	0.654^c^	0.539^b^	0.386^c^
Nasal ChT (μm)	175.1 ± 73.7	180.8 ± 72.5	168.8 ± 53.8	0.591^c^	0.732^b^	0.511^c^
Subfoveal ChT (μm)	268.8 ± 84.6	286.2 ± 80.7	266.2 ± 51.8	0.307^c^	0.896^b^	0.303^c^
Temporal CVI (%)	61.6 ± 4.5	64.1 ± 3.0	65.8 ± 2.4	**0.003^c^**	**<0.001^b^**	0.032^c^ ns
Nasal CVI (%)	61.0 ± 3.9	63.9 ± 4.4	64.2 ± 2.6	<0.001^*^	**0.002^a^**	0.614^*^
Subfoveal CVI (%)	64.5 ± 3.1	66.6 ± 1.8	67.5 ± 1.6	0.002^*^	**<0.001^a^**	0.111^*^

**Notes.**

* Mann–Whitney *U* test.

a Wilcoxon test.

c Independent sample *t*-test.

b Paired sample *t*-test.

Statistical significance was set at Bonferroni-adjusted *α* = 0.0167. *P* values < 0.0167 were considered significant.

ns not significant after correction NAION non-arteritic ischemic optic neuropathy ChT choroidal thickness CVI choroidal vascularity index

Bold values indicate statistically significant differences (*p* < 0.05).

Tables S1 and S2 present Cohen’s d effect sizes and 95% CIs for each parameter in the NAION (affected and unaffected eyes) and control groups. Compared with unaffected and control eyes, NAION eyes showed significant reductions in ONH pVD, RPC pVD, and RNFL thickness values. With regard to CVI measurements, NAION eyes exhibited medium effects for nasal (d = −0.97, 95% CI [−1.55, −0.38]) and temporal (d = −1.19, 95% CI [−1.76, −0.56]) CVI and a large effect for subfoveal CVI (d = −1.17, 95% CI [−1.76, −0.56]) when compared with unaffected eyes. Compared with control eyes, NAION eyes showed large and significant effects for temporal (d = −0.67, 95% CI [−1.09, −0.28]), nasal (d = −0.97, 95% CI [−1.55, −0.38]), and subfoveal (d = −1.17, 95% CI [−1.77, −0.56]) CVI. When unaffected eyes were compared with control eyes, only RPC temporal pVD showed a large effect (d = −1.03, 95% CI [−1.62, −0.44]), with medium effects observed for overall RPC pVD mean (d = −0.76, 95% CI [−1.33, −0.18]) and temporal CVI (d = −0.62, 95% CI [−1.19, −0.05]). All other parameters showed small, non-significant effects (Tables S1, S2).

For NAION eyes, weak positive correlations were observed between the mean ONH pVD and the mean RNFL thickness (*r* = 0.398/P = 0.049), while moderate positive correlations were observed between the mean RPC pVD and the mean RNFL thickness (*r* = 0.520/P =0.008), between RPC FA and the mean RNFL thickness (*r* = 0.483/P = 0.014), and between the mean CVI and mean ChT (*r* = 0.408/P = 0.043). For the unaffected eyes, moderate positive correlations were observed between RPC FA and the mean RNFL thickness (*r* = 0.477/P = 0.016) and between the mean CVI and mean ChT (*r* = 0.414/P = 0.040). No significant correlations were observed between the measurements for control eyes. However, following false discovery rate (FDR) correction for multiple comparisons, none of the initially observed correlations remained statistically significant (FDR-adjusted *P* > 0.05) (Table 3).

**Table 3:** Correlation analysis among parameters within each group.

	**Mean RNFL**		**Mean ChT**		**Mean CVI**	
	r	P	r	P	r	P
*NAION eyes (n* = 25*)*						
Mean ONH pVD (%)	**0**.**398**	**0.049**	0.058	0.783	0.120	0.567
Mean RPC pVD (%)	**0**.**520**	**0.008**	0.120	0.566	0.129	0.539
ONH FA (μm2)	0.251	0.225	−0.073	0.728	−0.047	0.822
RPC FA (μm2)	**0**.**483**	**0.014**	0.193	0.356	0.266	0.198
Mean RNFL (μm)	1	N	0.055	0.794	0.098	0.641
Mean ChT (μm)	0.055	0.794	1	N	**0**.**408**	**0.043**
Mean CVI (%)	0.098	0.641	**0**.**408**	**0.043**	1	N
*Fellow Eyes (n* = 25*)*						
Mean ONH pVD (%)	0.295	0.152	−0.132	0.531	−0.034	0.871
Mean RPC pVD (%)	0.229	0.271	−0.209	0.305	−0.001	0.997
ONH FA (μm2)	0.377	0.063	−0.210	0.314	−0.101	0.630
RPC FA (μm2)	**0**.**477**	**0.016**	−0.149	0.476	−0.107	0.612
Mean RNFL (μm)	1	N	0.100	0.636	0.293	0.155
Mean ChT (μm)	0.100	0.636	1	N	**0**.**414**	**0.040**
Mean CVI (%)	0.293	0.155	**0**.**414**	**0.040**	1	N
*Control eyes (n* = 25*)*						
Mean ONH pVD (%)	0.143	0.495	−0.312	0.129	−0.169	0.419
Mean RPC pVD (%)	0.136	0.516	−0.257	0.215	−0.039	0.852
ONH FA (μm2)	0.103	0.626	0.062	0.770	−0.226	0.276
RPC FA (μm2)	−0.010	0.963	−0.256	0.216	−0.232	0.264
Mean RNFL (μm)	1	N	−0.164	0.434	−0.048	0.820
Mean ChT (μm)	−0.164	0.434	1	N	0.292	0.156
Mean CVI (%)	−0.048	0.820	0.292	0.156	1	N

**Notes.**

Spearman correlation analysis, *r*: correlation coefficient. Significant *P* values (<0.05) are indicated in bold.

False discovery rate (FDR) correction was applied across all 45 pairwise correlations (15 per group) to control for multiple comparisons.

Unadjusted *P* values < 0.05 are shown in bold. None remained significant after FDR correction.

NAION non-arteritic ischemic optic neuropathy FA flow area pVD peripapillary vessel density ONH optic nerve head RPC radial peripapillary capillary RNFL retinal nerve fiber layer thickness ChT choroidal thickness CVI choroidal vascularity index

**Figure 4:**
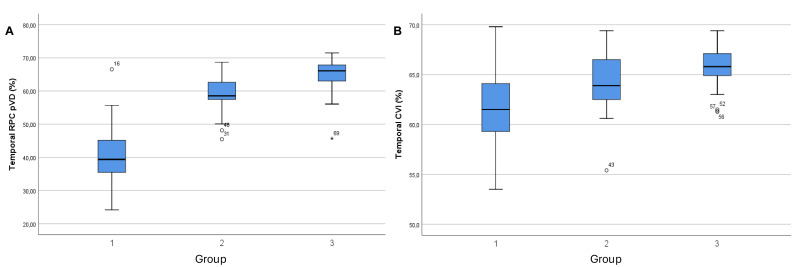
Group-wise comparison of temporal RPC vessel density and temporal CVI among NAION, unaffected fellow, and control eyes. Boxplots showing group-wise comparisons of (A) temporal radial peripapillary capillary (RPC) vessel density (pVD) and (B) temporal choroidal vascularity index (CVI) across NAION, unaffected, and control eyes. Boxes represent the interquartile range (IQR), horizontal lines indicat e median values, and dots represent individual data points.

As shown in Fig. 4, boxplots demonstrated a clear stepwise reduction in both temporal RPC pVD and temporal CVI, in the following order: control, unaffected, and NAION eyes. NAION eyes exhibited the lowest median values and the widest interquartile ranges; this indicated greater variability in microvascular impairment within this group. Unaffected eyes showed values intermediate to those for NAION and control eyes; this suggested subclinical hemodynamic compromise. Outliers observed for NAION eyes likely reflect interindividual variability in the severity of ischemic damage. These visualised trends support the presence of progressive vascular attenuation and regional susceptibility in the temporal peripapillary sector.

## Discussion

Our study found reductions in pVD in the RPC and ONH layers across all regions in NAION eyes compared with those in unaffected and healthy eyes, with the most significant reductions in temporal RPC pVD and the mean RPC pVD. These findings gain further significance when evaluated in light of normative datasets, which demonstrate high repeatability and low variation in pVD measurements assessed by OCTA in the healthy population (Durbin et al., 2017).

Our study also included a comparative analysis of affected and unaffected eyes, which provided insights into potential contralateral susceptibility. Compared with healthy eyes, unaffected eyes showed a significant decrease in pVD in the temporal region of the RPC layer. Haemodynamic imbalances likely affect these regions the most because of the smaller diameter and larger perfusion area of capillaries in the superior and temporal regions (Yu et al., 2019). Chou et al. (2022) found a decrease in pVD across all RPC segments in NAION eyes, compared with that in healthy and unaffected eyes, with the largest reductions occurring in the temporal quadrants of the ONH and RPC layers. Several studies have reported that, in chronic-phase patients, the superior and temporal peripapillary regions are more affected than are the inferior regions, compared with those in controls (Kaya, 2022; Pugazhendhi et al., 2022; Su et al., 2022). Additionally, Su et al. (2022) found that pVD in the superotemporal region had significant diagnostic accuracy in terms of distinguishing between NAION and healthy control groups. These vascular alterations could also be influenced by systemic vascular risk factors such as hypertension or diabetes mellitus or by anatomical crowding of the optic disc, both of which may predispose eyes to NAION.

From a clinical perspective, these findings emphasise the temporal peripapillary region as a critical site for early vascular compromise. Detection of reduced pVD in this area, even in clinically unaffected eyes, may help identify individuals at increased risk of NAION. Therefore, OCTA evaluation of the temporal RPC sector could serve as a valuable tool for early risk stratification and longitudinal monitoring.

Our results also showed a significant decrease in FA in the ONH and RPC layers in NAION eyes compared with those in both healthy and unaffected eyes. Furthermore, FA in the RPC layer was significantly lower in unaffected eyes than in healthy eyes, with no difference observed in the ONH layer between the two groups. These findings suggest that the RPC layer plays a critical role in NAION pathophysiology, likely because of its involvement in blood flow regulation. Moreover, the RPC layer is more severely affected by ischaemic events than is the ONH layer, as evidenced by the greater number of affected areas in the RPC layer. The ONH layer appears to be secondarily affected by NAION. Several factors may explain this. First, compared with other retinal capillaries, RPCs are longer and flatter and connect directly to the major venous trunk without anastomosing with arteries; thus, they are more sensitive to reduced blood flow. Second, metabolic demand may decrease due to a reduction in nerve fibres or impaired function (Liu et al., 2017). With the detailed identification of layer-specific vascular signals and anatomical boundaries with OCTA technology (Campbell et al., 2017), these results reflect the specific response of each retinal vascular layer to ischaemic events.

Compared with that in both healthy and unaffected eyes, RNFL thickness was significantly reduced in all sectors in NAION eyes, with no significant differences between the healthy and unaffected eyes. This structural loss confirms the well-known neurodegenerative consequence of ischaemic optic neuropathy. Previous studies have consistently linked abnormalities in pVD within the RPC layer to RNFL alterations in NAION (Arnold, 2003; Hondur & Budakoglu, 2022). In our initial analysis, RPC pVD showed a statistically significant positive correlation with RNFL thickness in NAION eyes; this reflected the biological dependence between structural and microvascular integrity. After FDR was applied, none of the correlations remained statistically significant; however, the association between RPC pVD and RNFL thickness in NAION eyes persisted at a trend level, which may still be clinically relevant. Collectively, our finding of widespread RNFL thinning, together with the instability of the pVD–RNFL correlation, suggests that RNFL thinning represents a late-stage consequence of the disease. Therefore, the RPC layer, which reflects early microvascular compromise rather than subsequent axonal degeneration, may serve as a more sensitive and earlier predictor of NAION than does RNFL thickness.

Previous studies comparing ChT between NAION and healthy eyes have yielded highly variable results (Fard et al., 2015; García-Basterra et al., 2016; Jiang et al., 2016). Jiang et al. (2016) found significantly greater ChT in NAION eyes during the acute phase, with no significant difference in the chronic phase. These findings suggest that peripapillary choroidal thickening in certain segments may be associated with optic disc oedema in the early stages of NAION. Other studies have suggested that choroidal thickening may reflect changes in the self-regulating capacity of the choroidal circulation following ischaemic events. In the acute phase, ChT may increase because of the enlargement of vascular components. This enlargement resolves as healing occurs, and ChT returns to normal. However, our study did not evaluate the acute phase and did not observe a significant difference in ChT.

Limited data are available on CVI in patients with NAION (Guduru et al., 2019; Pellegrini et al., 2019; Lu et al., 2022). Pellegrini et al. (2019) found no significant difference in CVI between acute NAION eyes and healthy controls, suggesting that NAION is not associated with choroidal hypoperfusion. In contrast, Guduru et al. (2019) reported significantly lower CVI in unilateral acute NAION and unaffected eyes than in healthy eyes. Similarly, Lu et al. (2022) found significantly lower CVI in NAION eyes (nine acute phase, eight non-acute phase) than in healthy eyes, with unaffected eyes also showing a significantly lower mean CVI.

In our study, CVI was significantly lower in three regions of NAION eyes than in those of healthy control eyes. This finding is consistent with the results of Guduru et al. (2019) and Lu et al. (2022).

Although a positive correlation between ChT and CVI was initially observed for both NAION and unaffected eyes, this association lost significance after FDR correction. This finding suggests a weak and non-specific relationship between these parameters. Similarly, Lu et al. (2022) reported a negative correlation between ChT and CVI only in unaffected eyes, while no significant correlation was detected in either acute or non-acute NAION eyes. These findings imply that the association between structural and vascular choroidal metrics may reflect physiological variations rather than a disease-specific mechanism.

Guduru et al. (2019) found a significant reduction in CVI within the temporal region of NAION eyes compared with that in unaffected eyes, whereas Lu et al. (2022) reported no significant differences. In contrast, our study observed a significant decrease in all quadrants of NAION eyes compared with those of unaffected eyes. These discrepancies may be attributed to variations in study populations, disease stages, and methods for acquiring and analysing OCT images.

While our results showed significant reductions in both OCTA parameters and choroidal parameters in affected eyes, no correlation was found between these two vascular metrics. This disparity is likely explained by the functional independence of the retinal and choroidal circulations, which reflects both distinct vascular plexuses and governing autoregulatory mechanisms. While OCTA assesses the retinal and ONH microvasculature, choroidal parameters more closely reflect deeper vascular layers supplied by the posterior ciliary arteries. The lack of correlation stems from the fundamental difference in regulation; the retinal autoregulation (governing RPC flow) is primarily responsive to local metabolic demands (neurovascular coupling), whereas the choroidal circulation (reflected by CVI) is predominantly influenced by autonomic nervous control and systemic pressure. Consequently, the fact that both parameters decreased but failed to correlate suggests that differential vascular responses of these tissues to ischaemia or autoregulatory failure may be at play. This disparity suggests that the highly vascularised choroid may adapt to chronic ischaemia, perhaps by relatively maintaining its integrity or adapting in a manner that does not parallel the microvascular damage observed in the RPC layer. Therefore, this lack of correlation underscores the need to treat these two vascular beds as independent systems in the pathophysiology of NAION. Further studies involving larger patient groups and varying disease stages are needed to better understand the underlying reasons for this functional disparity.

Guduru et al. (2019) observed significant reductions in both nasal and temporal CVI. Although the *P*-value for the difference in temporal CVI between unaffected and healthy eyes was below the conventional threshold (*P* = 0.032), it was not statistically significant after Bonferroni correction (adjusted *α* = 0.0167). The effect size for this comparison was moderate (Cohen’s d = −0.62, 95% CI [−1.19, −0.05]), indicating a potentially meaningful difference. This indicated that, despite not reaching statistical significance after correlation, the reduction in temporal CVI in unaffected eyes may still have clinical relevance.

The present study demonstrated that NAION significantly affected peripapillary vascular structures. The observed reductions in pVD and RNFL in both the ONH and RPC layers highlight the vulnerability of these areas to ischaemic damage The concurrent vulnerability of the temporal segment in both the RPC and CVI layers suggests regional susceptibility that could contribute to disease pathogenesis. However, no significant correlation was found between RPC temporal pVD and temporal CVI, indicating that retinal and choroidal microcirculatory changes may follow distinct pathophysiological pathways. Consequently, independent evaluation of these two compartments is essential for understanding NAION-related vascular alterations. Therefore, further prospective studies with larger sample sizes and standardised imaging protocols are necessary to validate our findings.

**Figure 5:**
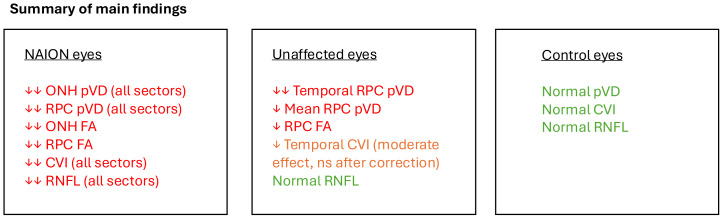
Summary schematic of the study’s main findings. A progressive decrease in peripapillary vessel density (pVD), flow area (FA), choroidal vascularity index (CVI), and RNFL thickness was observed from control to unaffected and NAION eyes. The unaffected eyes exhibited subclinical vascular compromise, particularly in the temporal RPC region, while the reduction in temporal CVI showed a moderate effect (Cohen’s *d* = 0.6) but did not remain significant after Bonferroni correction. Arrows indicate relative effect sizes (↓ medium, ↓↓ large).

These structural and vascular alterations are schematically summarised in Fig. 5, which illustrates the progressive reduction in RPC pVD, CVI, and RNFL thickness across the study groups. This schematic highlights the temporal sector as a region of particular vulnerability, even in clinically unaffected eyes, suggesting its potential role as a subclinical biomarker for NAION susceptibility.

Although our study provides valuable insights, it has certain limitations. As this was a retrospective study, we could not obtain data on factors, such as axial length, which could influence OCTA. Moreover, data on systemic comorbidities were not consistently available and were consequently not included in the analysis. Therefore, the potential influence of these systemic factors on retinal or choroidal perfusion parameters could not be fully evaluated. Additionally, although the images were converted to binary form using software, the possibility of overestimation or underestimation of both SA and LA remains. Furthermore, the retrospective, single-centre design of this study may limit the generalisability of the findings. However, the use of strict inclusion and exclusion criteria, along with the selection of age- and sex-matched control subjects, enhances the internal validity. Finally, as the measurements were obtained semi-automatically by a single examiner, a minor degree of measurement error or observer bias cannot be completely excluded. Therefore, the results may be applicable to similar patient populations with comparable clinical characteristics. Future prospective studies with larger sample sizes and standardised imaging protocols are needed to validate our findings and explore the temporal relationship between CVI changes and NAION progression.

## Conclusion

The study aimed to investigate the relationship between peripapillary and choroidal vascular parameters in NAION. The results showed significantly reduced pVD, RNFL thickness, and CVI in affected eyes compared to both unaffected and control eyes. Although correlations between peripapillary and choroidal parameters were not statistically significant in any group, this lack of correlation likely reflects distinct perfusion pathways and autoregulatory mechanisms between the retinal and choroidal circulations. These findings suggest that OCTA-derived measurements, particularly in the temporal peripapillary region, may serve as potential biomarkers for early detection and monitoring of vascular alterations in NAION. Further prospective studies are needed to determine whether the observed retinal and choroidal vascular differences represent independent pathogenic processes or sequential changes in disease progression. Furthermore, studies should investigate whether clinically relevant temporal RPC perfusion predicts the risk of NAION, along with variations in CVI by clinical stage.

## Supplemental Information

10.7717/peerj.20695/supp-1 Supplemental Information 1 Effect sizes (Cohen’s d) and 95% confidence intervals for intergroup comparisons of OCTA parameters

10.7717/peerj.20695/supp-2 Supplemental Information 2 Effect sizes (Cohen’s d) and 95% confidence intervals for intergroup comparisons of CVI

10.7717/peerj.20695/supp-3 Supplemental Information 3 STROBE checklist

10.7717/peerj.20695/supp-4 Supplemental Information 4 Complete Raw Data Used for Statistical Analyses

## Additional Information and Declarations

### Data Availability

The following information was supplied regarding data availability: The raw data supporting the findings of this study are available in the Supplemental File.
