# Supplementary material for: Optic disc parameters and choroidal vascular index as potential risk indicators in non-arteritic anterior ischaemic optic neuropathy: a retrospective study
Source: PeerJ. 2026 Jan 28;14:e20695. doi: 10.7717/peerj.20695 (PMC12860275; doi:10.7717/peerj.20695)
Supplement: Supplemental Information 1 [file peerj-14-20695-s001.docx]

| Supplementary Table S1:  Effect sizes (Cohen’s d) and 95% confidence intervals for intergroup comparisons of OCTA and structural parameters | | | |
| --- | --- | --- | --- |
|  | **NAION vs Control**  **d / %95 CI** | **NAION vs Unaffected**  **d / %95 CI** | **Unaffected vs Control**  **d / %95 CI** |
| **Mean ONH pVD (%)** | -2.71 [-3.49,-1.93] | -2.11 [-2.82,-1.39] | -0.53 [-1.09,0.03] |
| **Nasal ONH pVD (%)** | -2.32 [-3.40,-1.59] | -1.99 [-2.67,-1.3] | -0.27 [-0.83,0.28] |
| **Temporal ONH pVD (%)** | -1.98 [-2.65,-1.29] | -1.45 [-2.01,,-0.88] | -0.47 [-1.03,0.09] |
| **Mean RPC pVD (%)** | -3.25 [-4.09,-2.39] | -2.50 [-3.3,-1.69] | -0.76 [-1.33,-0.18] |
| **Nasal RPC pVD (%)** | -2.32 [-3.04,-1.6] | -2.02 [-2.71,-1.32] | -0.15 [-0.71,0.41] |
| **Temporal RPC pVD (%)** | -3.03 [-3.85,-2.21] | -2.03 [-2.71,-1.33] | -1.03 [-1.62,-0.43] |
| **Mean RNFL (μm)** | -2.00 [-2.68,-1.31] | -1.59 [-2.17,-0.98] | 0.27 [-0.29,0.82] |
| Effect sizes are reported as Cohen’s d with 95% confidence intervals.  NAION: non-arteritic ischemic optic neuropathy, FA: flow area, pVD: peripapillary vessel density, ONH: optic nerve head, RPC: radial peripapillary capillary RNFL: retinal nerve fiber layer thickness CVI: choroidal vascularity index | | | |
