# Supplementary material for: Optic disc parameters and choroidal vascular index as potential risk indicators in non-arteritic anterior ischaemic optic neuropathy: a retrospective study
Source: PeerJ. 2026 Jan 28;14:e20695. doi: 10.7717/peerj.20695 (PMC12860275; doi:10.7717/peerj.20695)
Supplement: Supplemental Information 2 [file peerj-14-20695-s002.docx]

| Supplementary Table S2:  Effect sizes (Cohen’s d) and 95% confidence intervals for intergroup comparisons of CVI | | | |
| --- | --- | --- | --- |
|  | **NAION vs Control**  **d / %95 CI** | **NAION vs Unaffected**  **d / %95 CI** | **Unaffected vs Control**  **d / %95 CI** |
| **Temporal CVI (%)** | -1.16 [-1.76,-0.56] | -0.67 [-1.09,-0.23] | -0.62 [-1.19,-0.05] |
| **Nasal CVI (%)** | -0.97 [-1.55,-0.38] | -0.93 [-1.39,-0.45] | -0.11 [-0.66,0.45] |
| **Subfoveal CVI (%)** | -1.17 [-1.76,-0.56] | -0.74 [-1.18,-0.29] | -0.5 [-1.06,0.06] |
| Effect sizes are reported as Cohen’s d with 95% confidence intervals.  NAION: non-arteritic ischemic optic neuropathy, FA: flow area, pVD: peripapillary vessel density, ONH: optic nerve head, RPC: radial peripapillary capillary RNFL: retinal nerve fiber layer thickness CVI: choroidal vascularity index | | | |
