# Supplementary material for: Optic disc parameters and choroidal vascular index as potential risk indicators in non-arteritic anterior ischaemic optic neuropathy: a retrospective study
Source: PeerJ. 2026 Jan 28;14:e20695. doi: 10.7717/peerj.20695 (PMC12860275; doi:10.7717/peerj.20695)
Supplement: Supplemental Information 3 [file peerj-14-20695-s003.docx]

STROBE Statement—checklist of items that should be included in reports of observational studies

|  | Item No | Recommendation |
| --- | --- | --- |
| **Title and abstract** | 1 | (*a*) Indicate the study’s design with a commonly used term in the title or the abstract  The study design is not explicitly stated in the title, but it is clearly indicated as a "retrospective analysis" in the abstract.  Abstract, line 19 |
|  |  | (*b*) Provide in the abstract an informative and balanced summary of what was done and what was found  The abstract provides a clear and balanced summary of the study design (retrospective cross-sectional), participant selection (25 NAION patients and 25 healthy controls), the imaging parameters assessed (OCTA-derived pVD, RNFL thickness, and CVI), and the key findings (significant reductions in OCTA parameters in NAION eyes, positive correlations between RNFL and OCTA metrics, and no significant correlation between retinal and choroidal parameters). These findings suggest that OCTA may be a useful tool for assessing vascular changes in NAION.  Abstract, line 13-35 |
| Introduction | | |
| Background/rationale | 2 | Explain the scientific background and rationale for the investigation being reported  NAION is commonly associated with compromised perfusion of the short posterior ciliary arteries, which supply the peripapillary choroid and contribute to optic nerve head perfusion. The vascular relationship between the optic disc and peripapillary choroid may play a key role in its pathogenesis. Choroidal vascularity index (CVI) is a reliable parameter for quantifying choroidal vasculature. Previous studies using OCTA are limited, and there is a lack of comprehensive data examining the relationship between optic disc parameters and choroidal metrics. This study aims to address this gap.  Introduction, line 45-56 |
| Objectives | 3 | State specific objectives, including any prespecified hypotheses  Optical coherence tomography angiography (OCTA) is increasingly used to examine retinal and choroidal changes in conditions such as NAION, where vascular pathology plays a key role (Spaide et al., 2018).  NAION has been associated with occlusion of the short posterior ciliary arteries, which supply blood to the peripapillary choroid and contribute to the optic nerve head’s blood supply. Consequently, impaired perfusion of the peripapillary choroid may contribute to NAION development. Recent studies suggest that the choroid may be involved in various optic nerve disc pathologies. The choroidal vascular index (CVI) has been proposed as a reliable parameter of choroidal vascularity (Agrawal et al., 2020; Iovino et al., 2020).  Assessing the correlation between the retina and choroid may be important to assess disease progression and identify potential risk factors. This study aims to investigate the mechanisms of vascular changes in diseases such as NAION by examining the relationship between retinal and choroidal parameters and improving disease monitoring. The study also aimed to explore whether optic disc and choroidal parameters may serve as potential risk indicators in NAION.  Introduction, line 38-55 |
| Methods | | |
| Study design | 4 | Present key elements of study design early in the paper  This is a retrospective, comparative, observational study.  Materials & Methods, line 61 |
| Setting | 5 | Describe the setting, locations, and relevant dates, including periods of recruitment, exposure, follow-up, and data collection  This retrospective cross-sectional study was conducted at the Department of Ophthalmology, Faculty of Medicine, Kahramanmaras Sutcu Imam University, a tertiary referral center with advanced imaging capabilities. The medical records of patients diagnosed with unilateral non-arteritic anterior ischemic optic neuropathy (NAION) between September 2019 and September 2022 were reviewed. Data from follow-up visits performed approximately 2 months after the resolution of optic disc edema were included in the analysis. The control group comprised 25 age- and sex-matched healthy individuals who had undergone OCTA as part of routine examination and were free of ocular pathology.  Materials & Methods, line 61-68, 82-85, 88-90 |
| Participants | 6 | (*a*) *Cohort study*—Give the eligibility criteria, and the sources and methods of selection of participants. Describe methods of follow-up  *Case-control study*—Give the eligibility criteria, and the sources and methods of case ascertainment and control selection. Give the rationale for the choice of cases and controls  *Cross-sectional study*—Give the eligibility criteria, and the sources and methods of selection of participants  Patients diagnosed with unilateral NAION who had data available after resolution of optic disc edema (mean 2 months) were included. Exclusion criteria were bilateral optic disc edema, any type of retinopathy, glaucoma, history of ocular surgery, other optic neuropathies, and refractive error exceeding ±3.00 diopters. The control group comprised healthy subjects without ocular pathology, matched for age (±2 years) and sex, whose right eyes were included for analysis. These individuals were selected from patients who had undergone OCTA imaging for non-pathological reasons during the same study period. For all three groups, images with signal strengths below 50 on OCTA imaging, abnormal segmentation, or motion-related artifacts were excluded.  Materials & Methods, line 81-95 |
|  |  | (*b*) *Cohort study*—For matched studies, give matching criteria and number of exposed and unexposed  *Case-control study*—For matched studies, give matching criteria and the number of controls per case  Controls were matched to NAION patients based on age (±2 year) and sex. Each group included 25 participants.  Materials & Methods, line 92-93 |
| Variables | 7 | Clearly define all outcomes, exposures, predictors, potential confounders, and effect modifiers. Give diagnostic criteria, if applicable  In this study, the primary outcome variables were retinal nerve fiber layer (RNFL) thickness, peripapillary vessel density (pVD), flow area (FA), choroidal thickness (ChT), and choroidal vascularity index (CVI). The main exposure variable was the diagnosis of unilateral non-arteritic anterior ischemic optic neuropathy (NAION). Predictor variables included ONH pVD, RPC pVD, FA, and CVI measurements. To minimize potential confounding, age- and sex-matched healthy controls were used. Systemic disease data were not consistently available due to the retrospective design and were therefore excluded from the analysis.  The diagnosis of NAION was based on the presence of sudden, painless, monocular visual loss; presence of relative afferent pupillary defect; optic disc edema; altitudinal visual field defect; normal ESR and CRP levels; absence of symptoms or findings suggestive of giant cell arteritis; and normal neuroimaging results.  Materials & Methods, line 65-68 |
| Data sources/ measurement | 8* | For each variable of interest, give sources of data and details of methods of assessment (measurement). Describe comparability of assessment methods if there is more than one group  All variables of interest were obtained retrospectively from OCTA images recorded in the follow-up of patients. All imaging was performed using the same device (RTVue-XR Avanti, Optovue Inc., Fremont, CA, USA) and the same software version (AngioVue).  Peripapillary vessel density (pVD) and retinal nerve fiber layer (RNFL) thickness were measured automatically using segmentation in ONH and RPC modes. Choroidal thickness (ChT) was measured manually at temporal, nasal, and subfoveal points using enhanced depth imaging. Choroidal vascularity index (CVI) was calculated semi-automatically using ImageJ software according to previously published protocols. All measurements were conducted with the same equipment and methods across all groups to ensure comparability.  Materials & Methods, line 96-110 |
| Bias | 9 | Describe any efforts to address potential sources of bias  To minimize selection bias, all eligible patients were included consecutively based on predefined criteria. Control subjects were age- and sex-matched to reduce confounding. All OCT and OCTA measurements were performed using the same device and protocols to avoid measurement bias.  Materials & Methods, line 70-71, 92-93, 96-97 |
| Study size | 10 | Explain how the study size was arrived at  The sample size was calculated using G*Power software based on data from a comparable study in the literature. The power analysis indicated that with a significance level of α = 0.05 and a statistical power of 80% (β = 0.20), an effect size of 0.79 would require a minimum of 25 eyes per group.  Materials & Methods, line 78-81 |
| Quantitative variables | 11 | Explain how quantitative variables were handled in the analyses. If applicable, describe which groupings were chosen and why  In this study, quantitative variables were analyzed as continuous data. Parameters were described using mean, standard deviation, median, minimum, and maximum values. No categorical groupings were applied.  Materials & Methods, line 111-112 |
| Statistical methods | 12 | (*a*) Describe all statistical methods, including those used to control for confounding  Statistical analyses were performed using SPSS version 28.0 (IBM Corp., Armonk, NY, USA). The distribution of variables was assessed using the Kolmogorov–Smirnov test. Between-group comparisons were conducted using the independent samples t-test for normally distributed variables and the Mann–Whitney U test for non-normally distributed variables. For intra-subject comparisons between affected and fellow eyes, paired sample t-tests and Wilcoxon signed-rank tests were used to account for the natural correlation between the eyes of the same individual. Categorical variables were compared using the chi-square test. Spearman correlation analysis was used to evaluate associations between parameters. To reduce the risk of Type I error due to multiple comparisons, Bonferroni correction was applied. No additional methods were used to control for confounding, as the groups were matched for age and sex.  Materials & Methods, line 111-123 |
|  |  | (*b*) Describe any methods used to examine subgroups and interactions  No subgroup analysis was performed. |
|  |  | (*c*) Explain how missing data were addressed  No missing data were present. |
|  |  | (*d*) *Cohort study*—If applicable, explain how loss to follow-up was addressed  *Case-control study*—If applicable, explain how matching of cases and controls was addressed  *Cross-sectional study*—If applicable, describe analytical methods taking account of sampling strategy  This was a retrospective cross-sectional study, and participants were selected based on strict inclusion and exclusion criteria. Control participants were matched by age (±2 years) and sex.  Materials & Methods, line 70-71, 92-93 |
|  |  | (*e*) Describe any sensitivity analyses  There was no loss to follow-up or sensitivity analysis, as this is a retrospective cross-sectional study. |

Continued on next page

| Results | | |
| --- | --- | --- |
| Participants | 13* | (a) Report numbers of individuals at each stage of study—eg numbers potentially eligible, examined for eligibility, confirmed eligible, included in the study, completing follow-up, and analysed  After applying inclusion and exclusion criteria, 25 patients were included in the NAION group. For each patient, the unaffected fellow eye was also analyzed. Additionally, 25 age- and sex-matched healthy controls were included. All participants had complete imaging data and were included in the final analysis.  Results, line 125-127 |
|  |  | (b) Give reasons for non-participation at each stage  Among the initially reviewed records of patients diagnosed with unilateral NAION, only those with complete imaging data and no exclusion criteria were included. Therefore, 25 eligible patients were included, and no participants dropped out during data collection or analysis.  Results, line 127-128 |
|  |  | (c) Consider use of a flow diagram  A flow diagram was not included; however, the study selection and analysis process is clearly described in the methods. A total of 25 eligible unilateral NAION patients were included after exclusion criteria were applied, along with 25 healthy controls, resulting in a total of 75 eyes analyzed.  Materials & Methods, line 81-95 |
| Descriptive data | 14* | (a) Give characteristics of study participants (eg demographic, clinical, social) and information on exposures and potential confounders  The study included three groups, each comprising 25 eyes: affected eyes of patients with unilateral NAION, their unaffected fellow eyes, and age- and sex-matched healthy control eyes. The mean age across groups was approximately 59 years, with 13 female and 12 male participants in each group. There were no statistically significant differences in age or sex distribution among the groups. Potential confounding variables such as systemic diseases were not included due to the retrospective nature of the data. Patients with a history of ocular surgery, significant refractive error (±3D), or other optic disc pathologies were excluded.  Results, line 128-130, Materials & Methods, line 86-88 |
|  |  | (b) Indicate number of participants with missing data for each variable of interest  There were no missing data for the variables of interest. |
|  |  | (c) *Cohort study*—Summarise follow-up time (eg, average and total amount) |
| Outcome data | 15* | *Cohort study*—Report numbers of outcome events or summary measures over time |
|  |  | *Case-control study—*Report numbers in each exposure category, or summary measures of exposure |
|  |  | *Cross-sectional study—*Report numbers of outcome events or summary measures  This information is presented in detail in the Results section and summarized in table 1, table 2, table 3 and table 4. Mean values, standard deviations, and p-values for key outcome variables (pVD, RNFL, CVI, ChT) are reported for each group.  pVD values, FA, and RNFL thickness were significantly lower in all quadrants in NAION eyes than in the other groups. The unaffected NAION eyes displayed significantly lower RPC mean, RPC temporal, and RPC FA compared to healthy control eyes (P= 0.011, P < 0.001, and P = 0.002, respectively). No significant differences were observed in optic nerve head (ONH) pVD, ONH FA, and RNFL between the unaffected and control eyes (Table 1).  The NAION eyes showed significantly lower CVI in the temporal, nasal, and subfoveal regions than the unaffected and control eyes. However, there were no significant differences in temporal, nasal, or subfoveal ChT between the NAION, unaffected, and control eyes. Additionally, no significant differences in CVI and ChT were observed between the unaffected and control eyes (Table 2).  Table 3 presents Cohen’s d effect sizes and 95 % CIs for each parameter across the three groups. NAION eyes showed significant reductions in ONH pVD, RPC pVD, and RNFL thickness compared to unaffected and control eyes. In CVI measurements, NAION eyes exhibited medium effects for nasal (d = –0.97, 95 % CI [–1.55, –0.38]) and temporal CVI (d = –1.19, 95 % CI [–1.76, –0.56]), and a large effect for subfoveal CVI (d = –1.17, 95 % CI [–1.76, –0.56]) versus unaffected eyes. Compared to controls, NAION eyes showed large and significant effects in temporal (d = –0.67, 95 % CI [–1.09, –0.28]), nasal (d = –0.97, 95 % CI [–1.55, –0.38]), and subfoveal CVI (d = –1.17, 95 % CI [–1.77, –0.56]). When comparing unaffected to control eyes, only RPC temporal pVD showed a large effect (d = –1.03, 95 % CI [–1.62, –0.44]), with medium effects observed for overall RPC pVD mean (d = –0.76, 95 % CI [–1.33, –0.18]) and temporal CVI (d = –0.62, 95 % CI [–1.19, –0.05]). All other parameters showed small, non-significant effects (Table 3).  In NAION eyes, weak positive correlations were observed between mean ONH pVD and mean RNFL (r = 0.398/P = 0.049), and moderate positive correlations were observed between mean RPC pVD and mean RNFL (r = 0.520/P=0.008), RPC FA and mean RNFL (r = 0.483/P = 0.014), and mean CVI and mean ChT (r = 0.408/P = 0.043). In the unaffected eyes, moderate positive correlations were observed between RPC FA and mean RNFL (r = 0.477/P = 0.016) and between mean CVI and mean ChT (r = 0.414/P = 0.040). No significant correlations were observed between the measurements in the control eyes (Table 4).  Results, line 125-160, table 1, table 2, table 3, table 4 |
| Main results | 16 | (*a*) Give unadjusted estimates and, if applicable, confounder-adjusted estimates and their precision (eg, 95% confidence interval). Make clear which confounders were adjusted for and why they were included  In this study, between-group comparisons were presented using mean differences and Cohen’s d effect sizes with 95% confidence intervals. No additional adjustment for confounders was necessary, as the study groups were age- and sex-matched, minimizing potential confounding effects. To account for multiple comparisons, Bonferroni correction was applied, and the threshold for statistical significance was adjusted accordingly. Corrected p-values were reported where applicable.  Results 142-153, table 1, table 2, table 3 |
|  |  | (*b*) Report category boundaries when continuous variables were categorized  Continuous variables were not categorized; thus, no boundaries were defined. |
|  |  | (*c*) If relevant, consider translating estimates of relative risk into absolute risk for a meaningful time period  Relative risks were not translated into absolute risks due to the cross-sectional design. |
| Other analyses | 17 | Report other analyses done—eg analyses of subgroups and interactions, and sensitivity analyses  No subgroup analyses were performed, but correlation analyses were conducted within each group. |
| Discussion | | |
| Key results | 18 | Summarise key results with reference to study objectives  The study aimed to investigate the relationship between peripapillary and choroidal vascular parameters in NAION. The results showed significantly reduced peripapillary vessel density, RNFL thickness, and CVI in affected eyes compared to both unaffected and control eyes. Although correlations between peripapillary and choroidal parameters were not statistically significant in any group, significant associations were found between structural and vascular parameters within affected eyes. These findings suggest that OCTA-derived measurements may help identify disease-related vascular alterations in NAION, even though peripapillary and choroidal compartments may be affected differently.  Discussion, line 270-277 |
| Limitations | 19 | Discuss limitations of the study, taking into account sources of potential bias or imprecision. Discuss both direction and magnitude of any potential bias  This retrospective design limited access to clinical variables such as axial length and systemic comorbidities, which were excluded due to incomplete records. Additionally, the CVI measurements based on binary image conversion may have led to over- or underestimation. Prospective studies with standardized protocols are needed to confirm these findings.  Discussion, line 258-263 |
| Interpretation | 20 | Give a cautious overall interpretation of results considering objectives, limitations, multiplicity of analyses, results from similar studies, and other relevant evidence  In summary, this study demonstrates that NAION significantly affects the optic nerve head and peripapillary vascular structures. The observed reductions in pVD and RNFL in both the ONH and RPC regions highlight the vulnerability of these areas to ischemic damage. Notably, pronounced alterations in the temporal segment suggest that this region may serve as a potential risk zone in the development of NAION. Despite the observed decline in CVI, the lack of significant correlation between retinal and choroidal parameters implies that these two vascular systems may be influenced by distinct pathophysiological mechanisms. Our findings are consistent with previous research and support the utility of OCTA as a valuable tool for the early detection and monitoring of NAION. However, future prospective, multicenter studies with larger sample sizes are needed to enhance the generalizability of these results.  Discussion, line 247-257 |
| Generalisability | 21 | Discuss the generalisability (external validity) of the study results  The retrospective and single-center design of this study may limit the generalisability of the findings. However, the use of strict inclusion and exclusion criteria, along with the selection of age- and sex-matched control subjects, enhances the internal validity. Therefore, the results may be applicable to similar patient populations with comparable clinical characteristics.  Discussion, line 263-270 |
| Other information | | |
| Funding | 22 | Give the source of funding and the role of the funders for the present study and, if applicable, for the original study on which the present article is based  This research received no specific grant from any funding agency in the public, commercial, or not-for-profit sectors. |

*Give information separately for cases and controls in case-control studies and, if applicable, for exposed and unexposed groups in cohort and cross-sectional studies.

**Note:** An Explanation and Elaboration article discusses each checklist item and gives methodological background and published examples of transparent reporting. The STROBE checklist is best used in conjunction with this article (freely available on the Web sites of PLoS Medicine at http://www.plosmedicine.org/, Annals of Internal Medicine at http://www.annals.org/, and Epidemiology at http://www.epidem.com/). Information on the STROBE Initiative is available at www.strobe-statement.org.
